# The Kusamala Program for primary caregivers of children 6–59 months of age hospitalized with severe acute malnutrition in Malawi: study protocol for a cluster-randomized controlled trial

**DOI:** 10.1186/s13063-017-2299-3

**Published:** 2017-11-17

**Authors:** Allison I. Daniel, Meta van den Heuvel, Wieger P. Voskuijl, Melissa Gladstone, Mike Bwanali, Isabel Potani, Celine Bourdon, Jenala Njirammadzi, Robert H. J. Bandsma

**Affiliations:** 1 Centre for Global Child Health, Hospital for Sick Children, Toronto, Ontario Canada; 2 Division of Gastroenterology, Hepatology and Nutrition, Hospital for Sick Children, Toronto, Ontario Canada; 3 Department of Nutritional Sciences, Faculty of Medicine, University of Toronto, Toronto, Ontario Canada; 4 Division of Paediatric Medicine, Hospital for Sick Children, Toronto, Ontario Canada; 5 Department of Paediatrics, Faculty of Medicine, University of Toronto, Toronto, Ontario Canada; 6 Global Child Health Group, Emma Children’s Hospital, Academic Medical Centre, University of Amsterdam, Amsterdam, The Netherlands; 7 Department of Paediatrics and Child Health, College of Medicine, University of Malawi, Blantyre, Malawi; 8 The Childhood Acute Illness & Nutrition Network (CHAIN), Nairobi, Kenya; 9 Department of Women’s and Children’s Health, Institute of Translational Medicine, University of Liverpool, Liverpool, UK; 10 Moyo Nutritional Rehabilitation and Research Unit, Queen Elizabeth Central Hospital, Blantyre, Malawi; 11 Department of Biomedical Sciences, College of Medicine, University of Malawi, Blantyre, Malawi

**Keywords:** Severe acute malnutrition, Psychosocial stimulation, Nutrition, WASH, Child development, RCT

## Abstract

**Background:**

Severe acute malnutrition (SAM) is associated with high mortality rates and impairments in growth and development in children that do survive. There are complex nutritional, health, and behavioural risk factors involving severely malnourished children and their primary caregivers, requiring integrated intervention approaches.

**Methods:**

A cluster-randomized controlled trial at the Queen Elizabeth Central Hospital in Blantyre, Malawi will be conducted to evaluate the effectiveness of a 4-day hospital-based intervention programme directed at primary caregivers. This programme, titled the Kusamala Program, aims to improve developmental and nutritional outcomes in children with SAM. Up to six primary caregivers and their children will be enrolled to groups each week, which will be randomly allocated to intervention or comparison arms. The intervention package consists of interactive counselling on three modules: 1) nutrition and feeding; 2) water, sanitation, and hygiene (WASH); and 3) psychosocial stimulation. Data collection will be performed at enrolment, at discharge from hospital, and at 6 months following discharge. The primary outcome is child development assessed with the Malawi Developmental Assessment Tool (MDAT), a validated measure of gross and fine motor, language, and social development.

**Discussion:**

This intervention programme is unique because it utilizes primary caregivers’ time spent in-hospital while children receive treatment for SAM. The programme has the potential to be effective in addressing multiple aspects of child, nutrition and development.

**Trial registration:**

ClinicalTrials.gov, NCT03072433. Registered on 7 March 2017.

**Electronic supplementary material:**

The online version of this article (doi:10.1186/s13063-017-2299-3) contains supplementary material, which is available to authorized users.

## Background

### The global burden of malnutrition

Worldwide, malnutrition is a direct or indirect cause of an estimated 45% of all child deaths [1]. Severe acute malnutrition (SAM) is one type of malnutrition that manifests in two forms: marasmus, identified by severe wasting; and kwashiorkor, characterized by the presence of bilateral pitting oedema [2]. Morbidities such as pneumonia, human immunodeficiency virus (HIV), or diarrheal disease often complicate effective management of SAM and are associated with increased mortality [1, 3–5]. Children with SAM are often admitted to hospital due to serious illness rather than malnutrition alone. Mortality in hospitalized children with SAM can be up to 35% even when World Health Organization (WHO) protocols are followed [4, 5].

### SAM and child development

Although most children survive episodes of SAM, there are limited studies that have examined long-term outcomes of SAM such as child growth and development. Children with SAM are known to be susceptible to nutritional problems such as growth faltering, and are hypothesized to have poor motor and cognitive outcomes due to maturation of the brain being constrained in malnourished children [4, 6–9]. The implications of impaired child development are major at the individual level due to reduced intellectual and physical capacity. The most prominent study on development in children with SAM was performed in a small cohort by Grantham-McGregor et al. beginning in 1975 [9, 13]. Results of this study suggested that children with SAM have long-term developmental delay compared to non-malnourished children [9, 13]. This can ultimately translate to decreased national-level economic progress [10–12].

### In-patient treatment guidelines for SAM

In-patient hospital treatment is required for children with SAM that have: 1) severe bilateral pitting oedema; 2) severe wasting with any bilateral pitting oedema or medical complications; 3) medical complications with any bilateral pitting oedema; or 4) no appetite [2, 14, 24]. The duration of treatment is generally between 1 and 2 weeks. The WHO *Guidelines for the inpatient treatment of severely malnourished children* include instructions to provide an environment that is stimulating for children during their treatment and support for primary caregivers, as well as preparation for follow-up after discharge from hospital with basic instructions for care of children [15].

Despite the promotion of psychosocial stimulation by the WHO, a recently published systematic review clearly outlines the need for scientific evidence behind the provision of psychosocial stimulation for children with SAM to improve developmental and nutritional outcomes [13, 16]. Only two trials, one in Jamaica by Grantham-McGregor et al. described previously and a second in Bangladesh, examined the effects of stimulation activities for children during hospital treatment of SAM [9, 13, 17]. In the Jamaican study, children with SAM were enrolled to either an intervention group (*n* = 21), receiving psychosocial stimulation 6 days per week in hospital and continuing weekly then bi-weekly over 3 years after hospital discharge, or to a comparison group (*n* = 18) [9]. These children were compared to non-malnourished controls (*n* = 21) [9]. This study, as well as the Bangladesh study, reported that psychosocial stimulation could help to recover some of the deficits in development in children with SAM [9, 13, 17]. Some of the limitations of these studies were a small sample size and a high likelihood for selection, reporting, and attrition bias. In addition, the developmental tools used were not locally standardized and the types of interventions tested in both studies are unlikely to be feasible in most resource-constrained settings [9, 13, 17].

### Trial and intervention rationale

In order to justify the implementation of psychosocial interventions for children with SAM, further research is needed. Importantly, there is evidence that links nutritional factors, water, sanitation, and hygiene (WASH), and psychosocial stimulation to child development and nutritional status [12, 18–20]. Integrated interventions that unite these elements could be most effective in improving developmental and nutritional outcomes in children with SAM.

## Methods

**Fig. 1:**
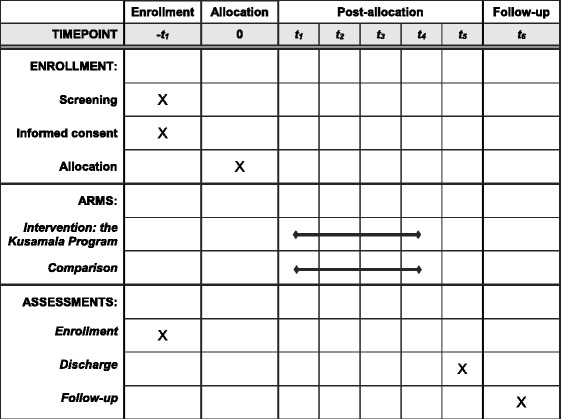
Timeline of enrolment, intervention and comparison, and assessments

The Standard Protocol Items Recommendations for International Trials 2013 Statement has been followed for the reporting of this protocol (see Additional file 1) and for reporting the timeline of the trial (Fig. 1) [21].

### Objectives and hypothesis

The primary objective of the trial is to evaluate the effects of a 4-day hospital-based intervention for primary caregivers of children with SAM on child development and nutritional status after 6 months.

The secondary objective is to understand the effects of this programme on knowledge, attitudes, and practices, as well as mental health status of primary caregivers.

The hypothesis is that the intervention programme for primary caregivers will improve developmental and nutritional outcomes in children hospitalized with SAM.

### Trial overview and design

This study will evaluate a comprehensive structured intervention package for primary caregivers of children aged 6 to 59 months hospitalized with SAM. It will be a pragmatic trial aimed at replicating how this type of intervention could be delivered in a real nutrition rehabilitation unit (NRU) setting and its effectiveness in this setting [22]. The primary outcome is child development, which will be assessed using the Malawi Developmental Assessment Tool (MDAT), a locally adapted and validated tool for evaluating multiple domains of child development (i.e. gross motor, fine motor, language, and social domains) [23].

The design of the study is a cluster-randomized controlled trial (superiority design) that will be conducted at the Moyo NRU of the Queen Elizabeth Central Hospital in Blantyre, Malawi (NCT03072433). A cluster-randomized controlled trial design was selected because the intervention is most feasible if delivered to groups of primary caregivers rather than individual caregivers. This design will also prevent temporal overlap between the two study arms: 1) an intervention arm of primary caregivers and children with SAM participating in an intervention programme; and 2) the comparison arm of primary caregivers and children with SAM receiving standard care. All other clinical and nutritional aspects of care will be equivalent; children will be treated per hospital protocol and WHO guidelines [24]. One day of each week will be used to recruit and enrol an estimated average of four primary caregiver-child pairs. Once formed, the group will be randomly assigned to either the intervention or comparison arm. Recruitment and enrolment will not overlap with the delivery of intervention sessions, although it is possible that children from different groups will be in the NRU at the same time depending on the duration of their in-patient treatment.

An internal pilot study (exploratory design) will be conducted within the full randomized trial and will reflect the design of the full trial [25, 26]. The internal pilot study will include at least the first 15 primary caregiver-child pairs recruited to each arm, for a minimum of 30 primary caregiver-child pairs [25, 26]. As there are few previous trials evaluating long-term development in children with SAM, this internal pilot study will inform the design of the larger randomized control trial by estimating the variance of MDAT scores. This will allow for a more accurate sample size calculation. The internal pilot study will also establish the adherence and engagement rates of participants to the intervention [25].

### Participant recruitment and enrolment

Children admitted to hospital with complicated SAM and their primary caregivers will be recruited at the NRU after being screened for eligibility within 3 days of admission to the NRU (*–t*_*1*_). It is estimated based on hospital records from the NRU that there will be sufficient SAM admissions to recruit primary caregivers and children to each group.

Prior to seeking informed consent, information about benefits and risks of this study will be provided verbally and on an information sheet in Chichewa to the primary caregivers of children admitted to hospital. Written informed consent will be requested of the primary caregivers on behalf of themselves and their children (*–t*_*1*_). For those unable to read or write, the information will be read to them in Chichewa. In these cases, informed consent by signature or thumbprint will be observed by an impartial witness.

### Identification of SAM in children

1. Marasmus or severe wasting, defined by weight-for-length *z* scores (WLZ) or weight-for-height *z* scores (WHZ) of at least three standard deviations (SD) below the median *or* by a mid-upper arm circumference (MUAC) of less than 115 mm; and/or
2. Kwashiorkor, defined by nutritionally-induced bilateral pitting oedema.

### Inclusion criteria

1. Child between 6 and 59 months with SAM;
2. Child admitted to hospital because of SAM with medical complications as defined by the current WHO guidelines or who has no appetite if there are no complications [2, 15];
3. Primary caregiver (self-identified) present at hospital.

### Exclusion criteria

1. Primary caregiver declined to give informed consent;
2. Child with a known terminal illness (i.e. in the opinion of the treating physician the child is likely to die within 6 months);
3. Child requires a surgical procedure.

This study will not exclude children with cerebral palsy and other types of neurodisability as they represent a significant portion of admitted children with SAM and could benefit from the intervention.

### Intervention: the Kusamala Program

The Kusamala Program is a 4-day counselling programme, led by NRU nurses, for primary caregivers of children with SAM in an NRU setting. Kusamala means “to take care” in Chichewa. The programme incorporates multiple modules aimed at addressing the diverse causes and consequences of SAM. The modules include:

1. Nutrition and feeding: breastfeeding, complementary foods available in the country, safe food preparation, prevention, and early identification of malnutrition;
2. WASH: handwashing of caregivers and children’s hands with soap after defecation and before preparing food and feeding; and
3. Psychosocial stimulation: to promote sensitive and responsive parenting and to identify activities that the primary caregiver and child can do together, including speaking or singing, playing with toys, and looking at pictures and books.

Each of the 4 days (*t*_*1*_ *–t*_*4*_) of the Kusamala Program involves 45 min of counselling performed in the back bay of the NRU, which is separated from the rest of the ward by a wall. The first three sessions cover each of the three modules (i.e. one module per session), whereas the final day is a summary of all modules.

Materials for the first two modules of the Kusamala Program were based on adaptable nutrition and WASH communication and support materials from the United Nations Children's Fund (UNICEF) [27, 28]. Nutritional messages and materials for children above 2 years of age have been added since these UNICEF materials focus on infants and young children. Furthermore, there will be an additional emphasis on prevention and early identification of malnutrition.

For the psychosocial stimulation module on the third day of the intervention, the WHO Care for Child Development Package will be used to promote sensitive and responsive caregiving for age- or ability-appropriate play and communication activities [29]. Psychosocial stimulation is an integral component of the Kusamala Program. For this reason, primary caregivers and their children are also involved in 45 min of supervised and interactive play on each of the 4 days after the counselling sessions. A basket of play items is available in the NRU for children and primary caregivers to use during the play sessions. There are traditional Malawian toys, including small drums, rattles, and shakers, in addition to “Western” toys, such as colourful stacking blocks. Primary caregivers can also make their own toys such as shakers by adding beans or bottle caps to empty plastic jars available in the basket. Nurses supervise these sessions and actively encourage primary caregivers to engage in play and communication activities appropriate for the age and stage of development of their children.

Four behaviour change techniques including information, materials, media, and performance have been integrated into the Kusamala Program. Results from 24 studies to improve health and nutritional outcomes showed that effect sizes on outcomes were higher when three or four techniques were applied [30]. Firstly, nurses use a flipbook with coloured images to provide information to participants [30]. Primary caregivers will be given: two take-home images of nutrition and WASH messages, respectively (i.e. media); a toy that is appropriate for their child’s stage of development (i.e. materials); and a certificate of completion of the Kusamala Program [30]. During the intervention sessions, nurses also involve primary caregivers in practicing relevant activities including feeding, handwashing, and play (i.e. performance) [30].

### Comparison: the standard of care and unsupervised play sessions

Participants in the comparison group will receive the standard care at the NRU including clinical stabilization and nutritional rehabilitation. Besides this, nurses will instruct primary caregivers to take their children to the area of the NRU where play items will be available for use during their hospital stay. However, nurses will not be involved in the same interactive psychosocial stimulation sessions in which participants in the intervention groups partake. Participants will not be counselled about the importance of play; primary caregivers will not be advised which toys are appropriate and the play sessions will not be supervised. Having primary caregivers and children go to the back bay also acts as a method of blinding study personnel and participants so they are unaware of the allocation.

At the time of discharge from the NRU, nurses counsel primary caregivers on nutrition and WASH for an estimated 15 min. Nurses have been trained on nutrition and WASH messages to discuss with participants at discharge, yet this counselling does not follow a strict protocol.

### Training of NRU nurses

Five Moyo NRU nurses were trained to conduct the Kusamala Program. Prior to the training they were given a test based on the Food and Agriculture Organization *Guidelines for assessing nutrition-related knowledge, attitudes and practices* to determine their baseline understanding [31]. Questions from the following modules were used: feeding young children (6–23 months), malnutrition, personal hygiene, water and sanitation, and food-based dietary guidelines [31]. Mean scores out of a possible 25 points were 21.8 ± 0.8, indicating that nurses already had familiarity of nutrition and WASH components as expected considering their work in the NRU. Nurses then received training for a duration of 6 hours and, over the next 2 weeks following training, the nurses practiced at least two sessions with primary caregivers and children in the ward. Nurses were then given the same test; mean scores were 24.2 ± 1.3. Differences between the pre-test and post-test were significantly different, showing an improvement in mean scores of 2.4 points (*p* = 0.02). Refresher training of NRU nurses will be conducted bi-annually. In addition, nurses received training in care for child development.

### Primary outcome

The primary outcome is child development 6 months after discharge (*t*_*6*_). Child development will be assessed with the MDAT. The MDAT has been evaluated in 1426 healthy Malawian children as well as children with neurodisability (*n* = 80) or with severe wasting (*n* = 120) [23]. In this evaluation, sensitivity was 97%, and 18% of healthy children failed, translating to specificity of 82% [23]. These results show that the MDAT is an appropriate tool for assessing child development in Malawi.

The MDAT will be used at discharge (*t*_*5*_) and 6 months later (*t*_*6*_) to examine development in four domains: gross motor, fine motor, language, and social development [23]. Children will be given a pass or fail depending on their ability to complete increasingly advanced items on the MDAT [23]. The sum of passed items can be calculated and translated to age-specific *z* scores based on healthy Malawian children as reference standards [23].

### Secondary outcomes

Data will be collected at three time points: at enrollment (–*t*_*1*_), discharge from hospital (*t*_*5*_), and 6 months after discharge (*t*_*6*_). All outcomes and measures are described in Table 1.

**Table 1:** Primary, secondary, and implementation outcomes and measures

Outcomes	Measures
Primary outcome	
Child development: gross motor, fine motor, language, and social development (*t*_*6*_)	Malawi Developmental Assessment Tool (MDAT)
Secondary outcomes (child)	
Nutritional status (*t*_*6*_)	Mid-upper arm circumference (MUAC) Weight-for-length or -height *z* scores (WLZ or WHZ) Presence of bilateral pitting oedema Height-for-age *z* scores (HAZ)
Dietary intake (*t*_*6*_)	24-h dietary recall
Readmission to hospital (*t*_*5*_–*t*_*6*_)	Record of readmission to hospital for severe acute malnutrition (SAM)
Mortality (*–t*_*1*_ to *t*_*5*_ or *t*_*5*_–*t*_*6*_)	Death of child confirmed
Secondary outcomes (primary caregiver and household)	
Mental health status (*t*_*6*_)	Self-Reporting Questionnaire-20 (SRQ-20)
Nutritional status (*t*_*6*_)	Mid-upper arm circumference (MUAC) Body mass index (BMI)
Knowledge, attitudes, and practices (*t*_*6*_)	Questionnaire about hygiene and sanitation, nutrition and feeding, and malnutrition 24-h dietary recall of child
Stimulus and support for children in the household (*t*_*6*_)	Home Observation of the Measurement of the Environment (HOME) Inventory
Implementation outcomes	
Participant engagement (*t*_*1*_)	Number of enrolled participants who attend day 1 of the Kusamala Program
Participant adherence (*t*_*1*_–*t*_*4*_)	Number of enrolled participants who attend all 4 days of the Kusamala Program
Fidelity of the intervention programme	Assessment form of Kusamala Program delivery
Participant knowledge and attitudes of intervention content (*t*_*5*_)	Questionnaire about hygiene and sanitation, nutrition and feeding, and malnutrition

Important child outcomes are the nutritional status assessed by anthropometry (i.e. MUAC, WLZ or WHZ, oedema, and height-for-age *z* scores) and dietary intake based on a locally adapted 24-h dietary recall which will be performed only at follow-up (*t*_*6*_) [32, 33]. Readmission to hospital and mortality at any time point are other secondary outcomes of interest. Child co-variates that will be considered include sex, age, and HIV status.

There are important co-variates in relation to households and primary caregivers measured at enrolment (–*t*_*1*_) that could influence the ability of primary caregivers to care for their children during hospital treatment and in the home, and both are known to influence child outcomes [12, 18, 34]. First, the mental health status of primary caregivers will be measured with the Self-Reporting Questionnaire, which has been translated and validated for use in Malawi [35, 36]. Maternal depressive symptoms have been shown to be significantly associated with adverse outcomes in infants such as cognitive and socioemotional problems [12, 18, 34]. Furthermore, nutritional status of primary caregivers according to two anthropometric outcomes, body mass index and MUAC, will be assessed. Other measured co-variates that could influence child outcomes include HIV status, age, education level, and marital status of the primary caregiver as well as location (i.e. urban or rural) and monthly income of the household, for example.

Primary caregiver outcomes of interest at 6 months (*t*_*6*_) will also include mental health status, nutritional status, as well as knowledge, attitudes, and practices relating to malnutrition, nutrition and feeding, and WASH based on the WHO *Indicators for assessing infant and young child feeding practices* [33]. Lastly, the environment in the home will also be assessed at follow-up 6 months after discharge (*t*_*6*_) using an adapted and translated version of the Home Observation of the Measurement of the Environment (HOME) Inventory [37]. The HOME Inventory includes observations and interview questions to the primary caregiver to establish the amount and quality of interaction and input in the child’s home environment; this may be related to developmental outcomes in children [11, 37].

### Implementation outcomes

Participant engagement and adherence to the Kusamala Program will be evaluated based on the proportion of participants that attend the first day of the programme (*t*_*1*_) and the number of participants that attend all 4 days of the programme (*t*_*1*_–*t*_*4*_), respectively. Fidelity of 20% of the intervention sessions will be assessed using a tool designed to capture duration of sessions, delivery of specific intervention content, and counselling skills according to the WHO Care for Child Development Package [29]. Following the intervention, knowledge and attitudes of primary caregivers who participate in the Kusamala Program will be demonstrated with the use of a questionnaire at discharge from hospital and at follow-up.

### Sample size calculation

The sample size estimate is based on cross-sectional study data of development of children with SAM at discharge from the NRU using the MDAT [38]. Results from this study showed that, based on MDAT *z* scores, the variance for gross motor was 1.9 SD, for fine motor was 1.2 SD, and for language was 1.7 SD [38]. Therefore, the composite variance for those domains was 1.6 SD. This was used to calculate the sample size using α = 0.05 and 80% power, an estimated intracluster correlation coefficient of 0.001, and with an expected effect size of 0.5 using Cohen’s *d* [39, 40]. With this approach, the initial estimate of the sample size for the primary outcome is a minimum of 160 primary caregiver-child pairs per arm (*n* = 320). As discussed previously, an internal pilot study will be conducted to determine the variance of the primary outcome in the first 15 children in each study group at 6 months post-discharge from hospital which will be used to recalculate the sample size [25].

### Biostatistics procedure and blinding

A biostatistician (CB) completed the computer-generated random allocation sequence for each study week and placed the sequence in sealed envelopes to be opened at the start of each week by a nurse at the NRU. NRU staff (apart from NRU nurses) and those doing screening, recruitment, enrolment, and data collection will be blinded. There are no expected reasons for un-blinding of study team members to occur. Blinding of the analysis will be performed by a biostatistician with the use of a dummy variable to code for each group. A data monitoring committee will not be necessary to analyse results un-blinded during the trial because the intervention is of very low risk. Nonetheless, an interim analysis will be completed with the internal pilot study data.

### Data collection and management

Data collection will be performed by three different enumerators who have been trained on all relevant assessment tools and questionnaires, including the MDAT. Baseline data collection will take place over approximately 80-week-long cycles, depending on enrolment rates, with assessments performed at enrolment (–*t*_*1*_) and at discharge (*t*_*5*_). These data collections will be done privately in the NRU, either in the procedures room or in the back bay depending on availability of these locations. Home visits will be conducted for follow-up assessments 6 months after children are discharged from hospital (*t*_*6*_) over the course of 80 weeks. For participants who prefer not to be visited in the home, they will be asked to come back to the NRU for follow-up assessments.

Investigators will maintain medical and research records for this trial in compliance with regulatory and institutional requirements for the protection of confidentiality of subjects. All personnel with access to data and information related to this trial will take all reasonable precautions to maintain the confidentiality of information. Data will be collected using paper forms which will be kept in a locked filing cabinet and will be accessed only by the investigators. Data entry will be performed into a Research Electronic Data Capture database, which includes automated checks for data values with coded numbers [41]. De-identified data will be exported in a password-encrypted file.

### Statistical analysis

**Fig. 2:**
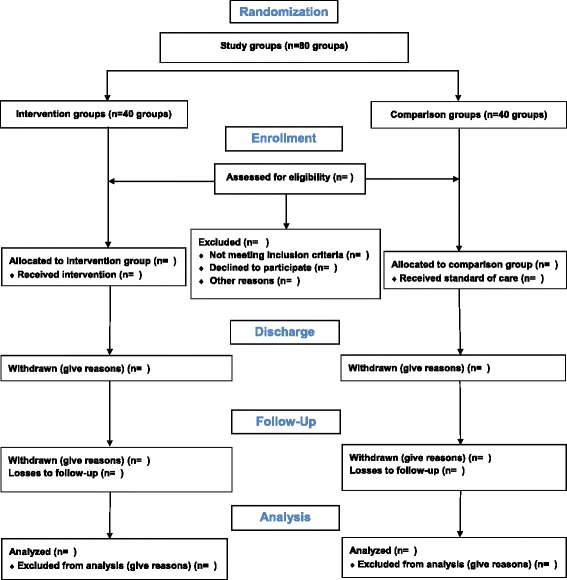
Modified version of the CONSORT 2010 flow diagram

Bias will be determined by maintaining a screening log of all potentially eligible participants and those who were excluded or recruited to the two arms. This will be presented as a flow diagram in the final report according to the Consolidated Standards of Reporting Trials (CONSORT) guidelines (Fig. 2) [42].

Data will be analysed using the statistical software Stata 14, R v3.2.5, and WHO Anthro v3.2.2 for anthropometric *z*-score calculations [43–45]. Missing data will be accounted for depending on the nature of drop-outs and loss to follow-up.

Descriptive statistics will be performed to summarize baseline characteristics of participants in both the intervention and comparison groups. More specifically, means and SD will be calculated for continuous variables such as the age of children, whereas the number of participants and percentages will be calculated for categorical variables such as the presence of oedema.

MDAT *z* scores standardized by age and based on a reference population of Malawian children will be calculated [23]. *Z* scores of each domain will be analysed separately to understand if the intervention influences certain domains more than others and a composite score of overall child development will be calculated if statistically appropriate. Upon completion of data collection for the internal pilot study, the variance of the primary outcome, child development using the MDAT, will be determined. The variance will be used to recalculate the sample size for the full trial [25, 26].

To determine the effect of the intervention on the primary outcome measures and continuous secondary outcomes, analysis of co-variance (ANCOVA) will be conducted to determine if there are differences between the intervention groups and the comparison groups while allowing for adjustment of co-variates in the analysis [46].

Multiple logistical regression will be performed to determine whether the intervention influenced binary outcomes such as the presence or absence of oedema. Subgroup analyses will be performed with participant age categories (i.e. infants and older children) and types of SAM (i.e. marasmus and kwashiorkor), and neurodisability, since children with neurodisability such as cerebral palsy are included in this study.

### Ethical considerations

NRU staff members involved in the trial, including nurses, will be taught about background information of the trial, scientific and ethical aspects of clinical research and trials, data collection tools, and communication to participants. Written consent for all participants will be taken by an enumerator after screening. All of these NRU staff members have completed Good Clinical Practice training and are fluent in Chichewa and English.

Participation in this study is completely voluntary for primary caregivers in the intervention and comparison groups. If primary caregivers are enrolled but choose to withdraw from the study at any time, they can do so without being asked to respond and without consequences. Confidentiality and respect of privacy of participants and those that withdraw will be maintained.

The Kusamala Program, which will be provided to participants in the intervention groups, is of very low risk and of potential benefit. The intervention package gives primary caregivers an opportunity to learn more about nutrition, health, and development of children. Adoption of caregiving practices requires behaviour change, which is dependent on the situation, motivation, and decisions of the primary caregivers. If primary caregivers choose to adopt recommended care practices, there may be positive outcomes for children involved in the study, other children in the household, and/or primary caregivers themselves.

Ethical approval for this study was obtained from the College of Medicine Research and Ethics Committee (P.04/16/1930) in Blantyre, Malawi, and the Research Ethics Board (1000053578) at the Hospital for Sick Children in Toronto, Canada. If any modifications are to be made to the study protocol, amendments will be submitted to both specified ethics boards and will be communicated to investigators and other study team members. It is expected that there will be no adverse events as a result of this low-risk intervention, yet if any adverse events do occur they will be recorded on a Hospital for Sick Children Adverse Event form within 48 h and will be a component of continuing review by the Hospital for Sick Children Research Ethics Board per the Tri-Council Policy Statement.

### Knowledge translation

Dissemination of results to the participants is difficult in this study primarily for logistical reasons. Firstly, it will take place in a low-resource setting with many participants living far from the NRU. Upon completion of the study, primary caregivers are unlikely to return to the hospital if their child is doing well.

Results of the trial with lessons learned will be communicated to colleagues at the NRU with the goal of transforming health services delivery for children admitted for in-patient treatment of SAM. Lessons learned and results will also be shared with collaborators and other international researchers through activities such as presentations at global health and nutrition conferences. To ensure that the study findings reach a broad audience, manuscripts will be submitted to open access peer-reviewed journals. Authorship will be granted for investigators and other personnel who play a significant role in the design and implementation of the intervention, data collection and management, analysis of results, or writing of the manuscript for this study. All authors will have access to the final trial dataset.

## Discussion

There is a need for evidence-based programmes that address nutritional, health, and behavioural factors that impact children with SAM and their families. This study is the first to examine the developmental and nutritional outcomes of a combined psychosocial stimulation, WASH, and nutrition programme in children with SAM. This type of intervention is highly relevant due to a growing focus on policy to improve child health, growth, and development, as indicated by the Sustainable Development Goals 2, 3, and 4, on reducing malnutrition, improving health and well-being, and promoting learning opportunities so children can achieve developmental milestones, respectively [47].

The Kusamala Program aims to improve outcomes in children with SAM by giving primary caregivers the knowledge and skills to enhance the care for their children. The Kusamala Program is unique in that it is a hospital-based intervention that matches the average minimum duration of in-patient treatment of SAM. In addition to ascertaining the effectiveness of the Kusamala Program to improve participant outcomes, this trial will also examine participant engagement and adherence and delivery of the Kusamala Program.

Although the Kusamala Program will first be implemented and evaluated in an NRU, the programme is novel and can also be adapted by other end-users for integration of psychosocial stimulation, nutrition, and WASH materials so that comprehensive programmes could be delivered in various contexts around the world such as refugee camps or other types of paediatric in-patient wards.

### Limitations

One potential limitation of the Kusamala Program is that it was designed to account for potential short lengths of hospital stay of study participants, and therefore does not cover all information in detail from the original counselling packages from the WHO and UNICEF [27–29]. Certain key messages were selected to be emphasized for the target population, yet not all topics will be covered because of these time constraints. A more complete intervention package of longer duration would potentially show greater improvements in developmental and nutritional outcomes; however, to reiterate, one of the benefits of the Kusamala Program is its feasibility and applicability for implementation in low-resource NRU settings.

Potential delays to the study are possible due to unforeseen circumstances that can suspend or extend the duration of the study. If there is a major event that delays the study, it is possible to postpone enrolment without causing disruption to the design or results. In this case, it would be important to still ensure that there is a 6-month period between discharge and follow-up for each group of the study to ascertain the effectiveness of the Kusamala Program as per the study protocol.

## Conclusion

The Kusamala Program was designed with the goal of improving developmental and nutritional outcomes in children with SAM. This cluster-randomized controlled trial is a methodologically sound approach to assessing the effectiveness of this intervention programme. By also examining implementation outcomes, including fidelity and participant engagement and adherence, delivery of the intervention and receptiveness of participants to the intervention, respectively, will also be recognized. If results from this cluster-randomized controlled trial are encouraging, the Kusamala Program can be adapted and implemented in other SAM treatment centres and beyond to reach as many primary caregivers and children as possible.

## Trial status

Recruitment of participants started on 28 November 2016 and is expected to continue until mid-2019.

## Additional file

Additional file 1: SPIRIT 2013 checklist. (DOC 122 kb)

## Abbreviations

ANCOVA: Analysis of covariance
CONSORT: Consolidated Standards of Reporting Trials
HIV: Human immunodeficiency virus
HOME: Home Observation of the Measurement of the Environment
MDAT: Malawi Developmental Assessment Tool
MUAC: Mid-upper arm circumference
NRU: Nutrition rehabilitation unit
SAM: Severe acute malnutrition
SD: Standard deviation
UNICEF: United Nations Children's Fund
WASH: Water, sanitation and hygiene
WHO: World Health Organization
WHZ: Weight-for-height *z* scores
WLZ: Weight-for-length *z* scores
